# Identification of Gallbladder‐Specific Distal Regulatory Sequence of Murine Sox17

**DOI:** 10.1111/gtc.13186

**Published:** 2024-12-26

**Authors:** Shihan Zeng, Ayaka Yanagida, Noriaki Ota, Mami Uemura, Yoshikazu Hirate, Ryuji Hiramatsu, Naoaki Mizuno, Yoshiakira Kanai, Masami Kanai‐Azuma

**Affiliations:** ^1^ Department of Veterinary Anatomy The University of Tokyo Tokyo Japan; ^2^ Research Center for Biological Products in the Next Generation National Institute of Infectious Diseases Tokyo Japan; ^3^ Department of Experimental Animal Model for Human Disease Institute of Science Tokyo Tokyo Japan; ^4^ Stem Cell Therapy Division Institute of Science Tokyo Tokyo Japan

**Keywords:** gallbladder, gene regulation, immunohistochemistry semiquantification, Sox17

## Abstract

*Sox17* is a key transcriptional regulator of endoderm formation and function in the gallbladder, blood vessels and reproductive organs. Although multiple transcript variants of *Sox17* have been suggested, the precise mechanisms underlying their time‐ and tissue‐specific expression remain unclear. In this study, we discovered two putative regulatory sequences (R1 and R2) adjacent to different transcription start sites of mouse *Sox17* exon 1 and generated deletion mice for these regions (*Sox17* ^*Δdr/Δdr*^ *)*. *Sox17* ^*Δdr/Δdr*^ mice were alive and fertile, and they possessed a normal‐sized gallbladder. However, semiquantitative analysis of immunostaining showed that the expression levels of SOX17 in *Sox17* ^*Δdr/Δdr*^ embryos were reduced to less than 50% of the wild‐type in the gallbladder epithelium. Furthermore, the bile ductal epithelium marker SOX9 was abnormally upregulated, and PAS/DBA‐positive mucin secretion‐like epithelial cells were induced in the *Sox17* ^*Δdr/Δdr*^ gallbladder. Our results demonstrate that the distal sequence of *Sox17*, including R1 and R2, is important for the regulation of *Sox17* gene expression in the embryonic gallbladder and is crucial for normal gallbladder epithelial development.

## Graphical abstract

*Sox17* regulates embryogenesis and tissue homeostasis, but the mechanisms of its specific expression remain unclear. In *Sox17* ^*Δdr/Δdr*^ mice, which lack a distal sequence with two putative regulatory regions, SOX17 expression is reduced in the gallbladder, with epithelial differentiation defects, highlighting the distal sequence's role in gallbladder development.

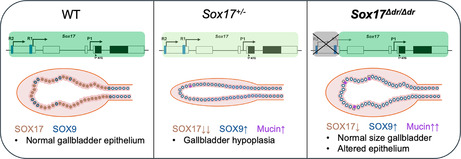

## 1 Introduction

The SOX family genes code transcription factors containing a high‐mobility group (HMG) box structure and show high homology to the mammalian sex‐determining gene *S* *ry*. More than 20 SOX family gene members have been identified from various species and classified into A–J groups in accordance with their genetic sequences and functional similarities (Bowles, Schepers, and Koopman 2000). They play critical roles in cell fate decisions and tissue differentiation during development. *Sox17*, which belongs to Group F, was identified as a master regulator of endoderm development (Kanai‐Azuma et al. 2002). *Sox17* null mice show midgut and hindgut endoderm deficiency, and they are embryonic lethal at approximately embryonic day (E)10.5. *Sox17* also regulates other organogenesis, such as the reproductive tract (Hirate et al. 2016; Uchida et al. 2022), cardiovascular system (Sakamoto et al. 2007), and gallbladder formation (Uemura et al. 2013). Rete testis‐specific *Sox17* conditional knockout male mice show a disrupted Sertoli valve, causing infertility (Uchida et al. 2022). Heterozygous *Sox17* mutant mice (*Sox17* ^*+/−*^) show decreased implantation rates (Hirate et al. 2016). In addition, SOX17 is expressed caudal to the hepatic diverticulum at E8.5 (Spence et al. 2009), which is essential for the development of the gallbladder and cystic duct (Uemura et al. 2013). *Sox17* haploinsufficiency reduced the proliferation of gallbladder epithelia, leading to gallbladder hypoplasia (Higashiyama et al. 2017; Pattarapanawan et al. 2020). Therefore, the expression level of SOX17 is crucial for embryonic development.

Understanding the regulatory mechanisms underlying *Sox17* gene expression and its function provides valuable insights the developmental biology and etiology of related congenital diseases. However, the mechanism by which the tissue‐ and time‐specific *Sox17* gene expressions are regulated in mice remains unclear. Tissue‐specific splicing variants of *Sox17* were identified in mice (Kanai et al. 1996). Later, several alternative promoters of *Sox17* during embryogenesis were suggested (Choi et al. 2012; Engert et al. 2009; Liao et al. 2009). Recently, the functions of two evolutionarily conserved putative promoter regions of *Sox17*, which are located upstream of the putative transcription start sites (TSSs), were investigated (Trinh et al. 2022). The deletion of the putative promoter adjacent to the TSS between exon 3 and exon 4 led to impaired vascular and endodermal development, causing embryonic lethality by E12.5. By contrast, the deletion of the other putative promoter adjacent to the TSS of exon 1 showed a modest increase in lympho‐vasculogenesis at E9.5. However, the functions of this region in other organs during late embryonic development and after birth remain unclear. Furthermore, although further distal TSS of *Sox17* has been suggested and *Sox17* variants with long forms of exon 1 were predicted in the Ensembl and UCSF databases, the importance of this distal regulatory sequence in *Sox17* remains unclear. In this study, the *Sox17* distal and proximal regulatory sequences of exon 1 deletion mice (*Sox17* ^*Δdr/Δdr*^) were generated to explore their roles in regulating SOX17 expression patterns and levels during embryogenesis and their impact on organogenesis.

## 2 Results

### 2.1 Conservation of the *Sox17* Gene and Its Putative Regulatory Sequences Across Animals

**FIGURE 1:**
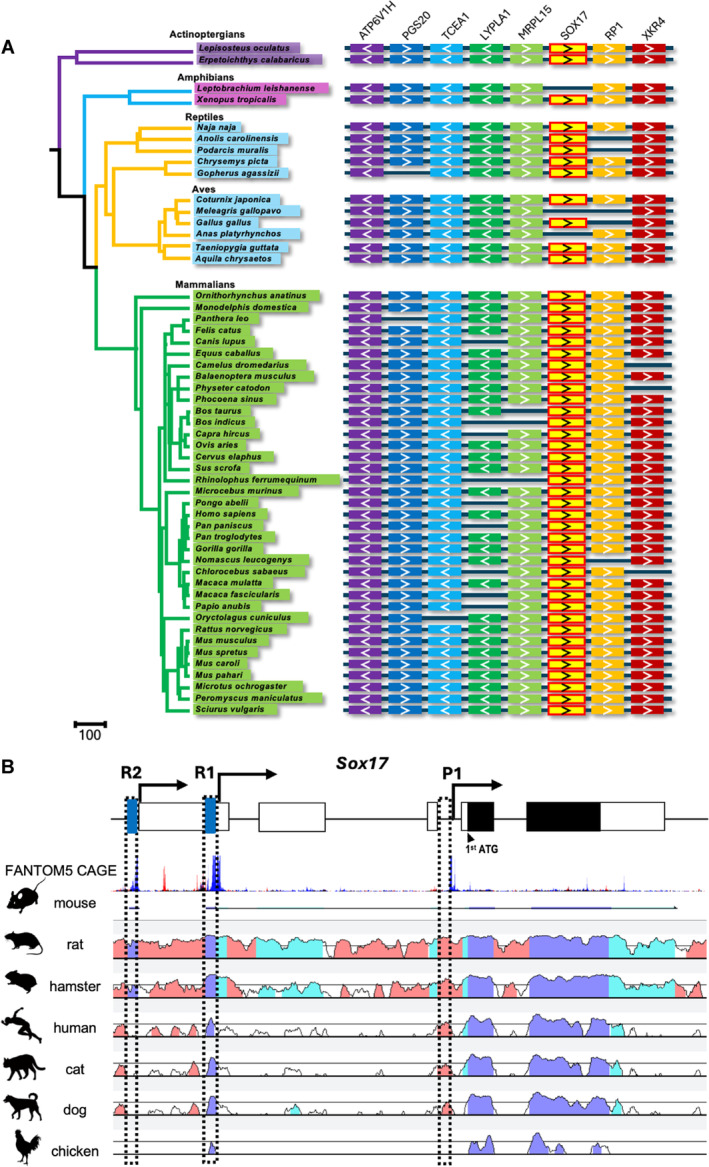
Conservation analysis of *Sox17*. (A) *Sox17* synteny conservation in vertebrates. Genes are depicted by colored boxes with an arrow. (B) FANTOM5 CAGE analysis of mouse *Sox17* and VISTA plots compared with other vertebrate orthologous regions. White box: Exons; black box: Protein‐coding regions. The dashed boxes of the putative regulatory sequences R1 and R2, as well as the reported promoter P1, are close to the TSS marked in FANTOM5 CAGE. Blue peaks: Total counts of CAGE read forward; red peaks: Total counts of CAGE read reverse; dark blue: Exons; light blue: UTRs; pink: Non‐coding regions.

To explore the evolutionary dynamics of the *Sox17* gene across vertebrates, synteny analyses of *Sox17* were performed using the available Ensembl genomic database (Figure 1A and Table S1). The *Sox17* gene and its upstream genes *Atp6v1h*, *Pgs20*, *Tcea1*, *Lypla1* and *Mrpl15*, as well as its downstream genes *Rp1* and *Xkr4*, were conserved as syntenic blocks in vertebrates.

In mice, multiple alternative promoters are suggested in *Sox17* (Engert et al. 2009; Liao et al. 2009). However, their locations and functions remain to be investigated. We, first, predicted the locations of the putative TSSs for murine *Sox*17 by analyzing the *Sox17* locus (chr1: 4,490,931‐4,497,354) using the FANTOM5 Cap Analysis Gene Expression (CAGE) database (Lizio et al. 2015). Several putative TSSs were predicted in the murine *Sox17* gene. We named the 150 bp regions adjacent to the putative TSSs as R1 and R2, apart from the recently confirmed putative promoter region (P1) located between exon 3 and exon 4 (Figure 1B). P1 and near the R1 region have been identified as a conserved putative promoter (Trinh et al. 2022). P1 deletion mice live longer than *Sox17* null mice, but they are embryonic lethal by E12.5 because of the impairment of vascular and endodermal development (Trinh et al. 2022). However, the functions of R1 and R2 in late embryonic development and after birth remain unknown. We compared the genomic sequences of R1 and R2 with those of rats, hamsters, humans, cats, dogs and chickens using the available datasets from Ensembl and Vista‐Point to examine the conservation of these regions among vertebrates (Frazer et al. 2004; Mayor et al. 2000) (Figures 1B and S1). The genomic sequence of R1 is highly conserved in birds and mammals, whereas that of R2 is predominantly conserved in mice, rats, and hamsters. This finding indicates that R2 was independently acquired by the suborder Myomorpha during evolution.

### 2.2 Generation of *Sox17* ^*Δdr*^ ^*/Δdr*^ Mice

**FIGURE 2:**
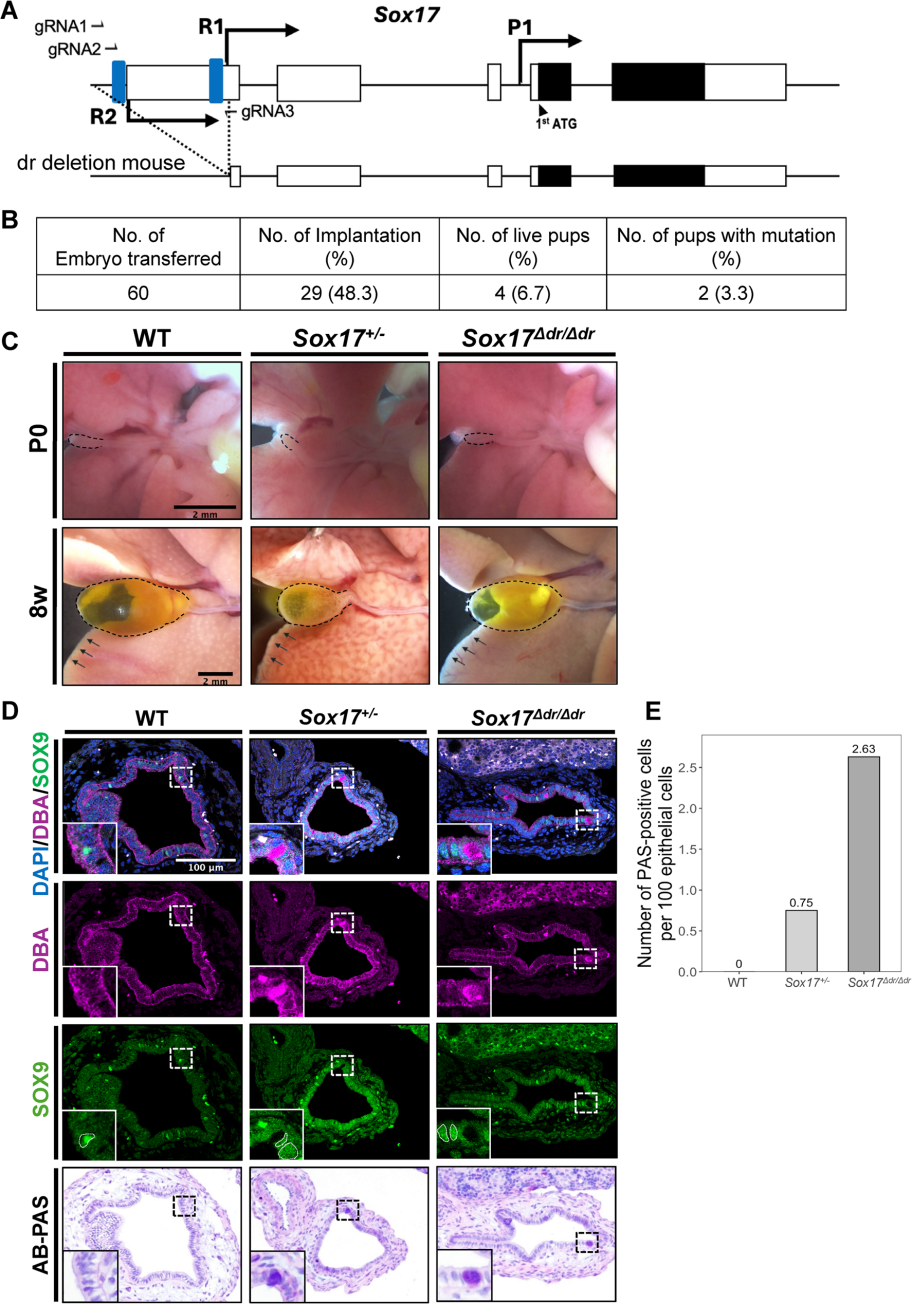
*Sox17* ^*Δdr/Δdr*^ mice show abnormal gallbladder differentiation. (A) Deletion strategy for generating *Sox17* ^*Δdr/Δdr*^ mice. (B) Developmental efficiency in zygote genome editing for *Sox17* distal regulatory sequence deletion. (C) The gallbladders and livers from P0 and 8‐week‐old WT, *Sox17* ^+/−^ and *Sox17* ^*Δdr/Δdr*^. Scale bars: 2 mm. The dashed lines and arrows show the outline of the gallbladder and liver. (D) Immunostaining and AB‐PAS staining of the P0 gallbladders of WT, *Sox17* ^+/−^ and *Sox17* ^*Δdr/Δdr*^ mice. The insets show the high magnification of the boxed regions. Scale bar: 100 μm. The dashed lines indicate the SOX9‐positive cells. (E) Comparison of the number of PAS‐positive gallbladder epithelial cells from WT, *Sox17* ^+/−^ and *Sox17* ^*Δdr/Δdr*^ at P0 (WT and *Sox17* ^+/−^ groups *n* = 4, *Sox17* ^*Δdr/Δdr*^ group *n* = 5).

To investigate the role of R1 and R2 in embryogenesis, we generated mice with the distal region deletion of *Sox17* exon 1 (*Sox17* ^*Δdr/Δdr*^), including the R1 and R2 regions, using CRISPR/Cas9 genome editing. We designed gRNAs upstream of the R2 region and downstream of the R1 region (Figures 2A and S2A). Two of the four live pups obtained mutation (Figure 2B). One of these male mutant mice (mutant‐1) was intercrossed with wild‐type (WT) mice to produce *Sox17* ^*Δdr/+*^ mice with the deletion of the distal putative regulatory sequences of *Sox17*, including R1 and R2. We crossed *Sox17* ^*Δdr/+*^ females from the F1 generation onwards with *Sox17* ^*Δdr/+*^ males from the F1 generation onwards. *Sox17* ^*Δdr/Δdr*^ mice developed to adulthood, although *Sox17* null mice are embryonic lethal (Kanai‐Azuma et al. 2002).

In addition, the impact of deleting the distal regions of *Sox17* on its mRNA expressions was assessed by RT‐qPCR. Given the technical limitation in isolating a small number of *Sox17*‐expressing cells from embryonic tissues, mRNA was analyzed from the whole gallbladders of E13.5 WT and *Sox17* ^*Δdr/Δdr*^ embryos (Figure S2B). *Sox17* mRNAs containing exon 1 and exon 2 or exon 1 and exon 3 were expressed. The expression level of *Sox17* mRNA containing exon 4 and exon 5, including the HMG box domain, was decreased in *Sox17* ^*Δdr/Δdr*^ gallbladder. These results indicate that transcripts from distal TSSs are expressed in WT embryos, and their expression was deleted in *Sox17* ^*Δdr/Δdr*^ embryos.

Other *Sox17*‐expressing organs were also assessed: the rete testes, oviduct and uteri. Rete testis‐specific *Sox17* conditional knockout male and *Sox17* ^*+/−*^ female mice show subfertility (Hirate et al. 2016; Uchida et al. 2022). However, the 8‐week‐old *Sox17* ^*Δdr/Δdr*^ testes did not show significant morphological abnormalities, and SOX17 was expressed in the rete testes similar to that in WT male mice (Figure S2C). In 3‐week‐old *Sox17* ^*Δdr/Δdr*^ females, which had not started the oestrous cycle, SOX17 was expressed in the luminal and glandular epithelium of the uteri, which is similar to WT female mice (Figure S2D). Males and females of *Sox17* ^*Δdr/Δdr*^ mice were fertile. These results indicate that the distal regions of *Sox17* exon 1 did not alter the male and female reproductive functions.

### 2.3 Abnormal Mucin Accumulation in the Postnatal *Sox17* ^*Δdr*^ ^*/Δdr*^ Gallbladder Epithelium

*Sox17* haploinsufficiency leads to gallbladder hypoplasia and causes biliary atresia with hepatic damage in the peripheral part of the liver (Pattarapanawan et al. 2020). However, postneonatal (P)0 and 8‐week‐old *Sox17* ^*Δdr/Δdr*^ mice did not show gallbladder hypoplasia or liver damage (Figure 2C). Thus, the detailed morphology of the *Sox17* ^*Δdr/Δd*^ gallbladder epithelium was further examined by histological analysis. The gallbladder epithelium secretes mucin into the lumen to protect itself from highly concentrated bile acid exposure. In the P0 WT, *Sox17* ^*+/−*^ and *Sox17* ^*Δdr/Δdr*^ gallbladders, the apical surface of the epithelium was positive for *Dolichos biflorus* agglutinin (DBA) lectin. However, a small number of cytoplasmic DBA‐positive cells were scattered in *Sox17* ^*+/−*^ and *Sox17* ^*Δdr/Δdr*^ gallbladder epithelium (Figure 2D). SOX9 (ventral pancreas and gallbladder primordia marker) was ectopically expressed in P0 *Sox17* ^*+/−*^ and *Sox17* ^*Δdr/Δdr*^ gallbladder epithelium. However, these cytoplasmic DBA‐positive cells were negative for SOX9. Alcian blue‐periodic acid Schiff (AB‐PAS) staining was also performed to examine glycogen accumulation. Serial section staining showed that cytoplasmic DBA‐positive cells were also positive for PAS, similar to goblet cells, which exist in the epithelium of the intestine and the common bile duct near the duodenum (Figure S2E). The number of PAS‐positive gallbladder epithelial cells in *Sox17* ^*Δdr/Δdr*^ mice was 2.63 times higher than that in WT mice (Figure 2E).

### 2.4 Downregulation of SOX17 Expression in *Sox17* ^*Δdr*^ ^*/Δdr*^ Mice Embryonic Gallbladder

**FIGURE 3:**
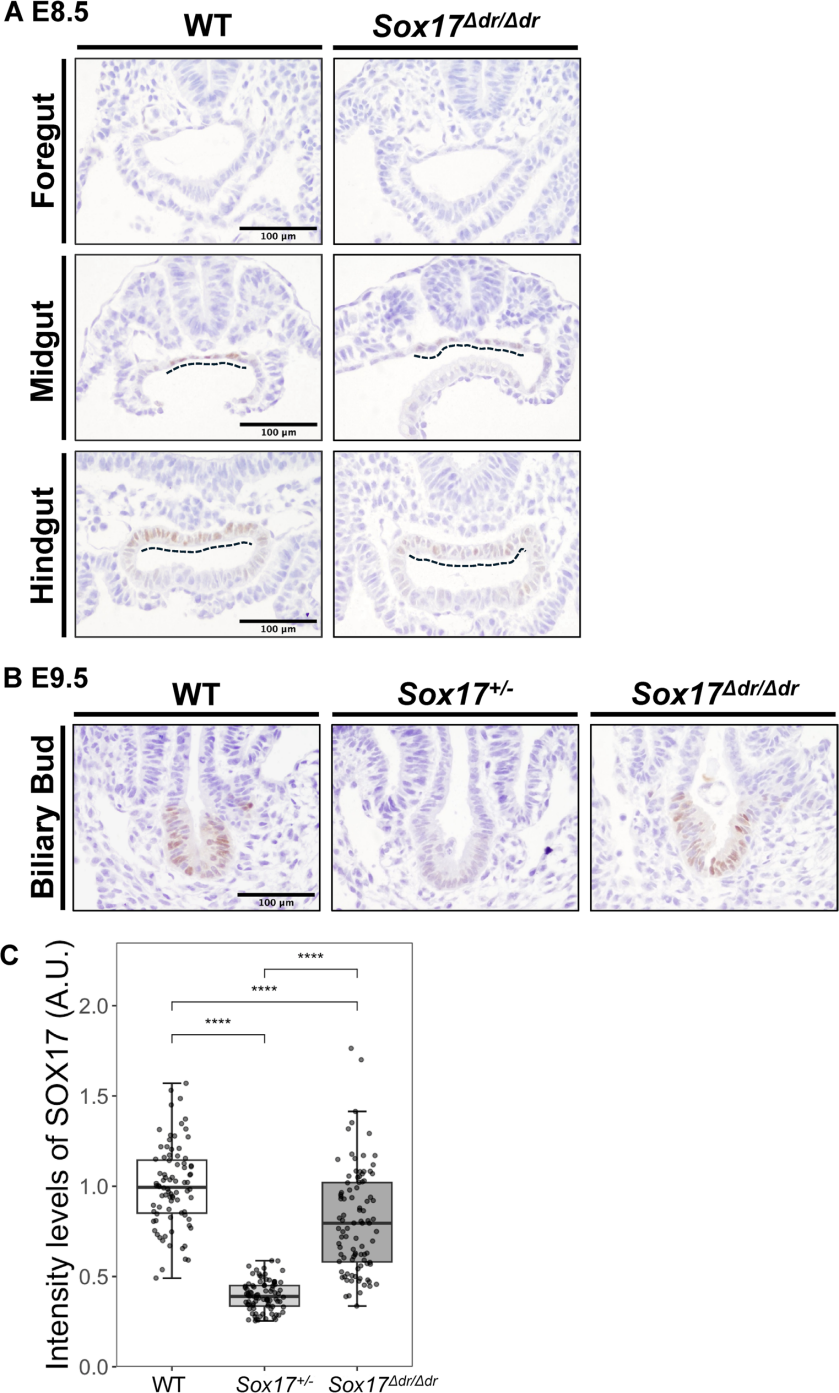
SOX17 expression in E8.5 and E9.5 *Sox17^Δ^* ^*dr/Δdr*^ mice. (A) Immunostaining of foregut, midgut and hindgut from WT and *Sox17* ^*Δdr/Δdr*^ mice at E8.5. The dashed line indicates SOX17 expression in the endoderm. Scale bars: 100 μm. (B) Immunostaining of the biliary duct from WT, *Sox17* ^+/−^ and *Sox17* ^*Δdr/Δdr*^ mice at E9.5. Scale bars: 100 μm. (C) Semiquantitative analysis of the intensity of SOX17 staining in the gallbladders at E9.5 of WT, *Sox17* ^+/−^ and *Sox17* ^*Δdr/Δdr*^. *n* = 4 for each group. The staining intensities were normalized to the average staining intensity of WT. ****: *p* < 0.0001.

To address when these abnormal gallbladder epithelium emerge, we examined SOX17 expression patterns during embryogenesis. The endoderm is patterned along the anterior–posterior axis into the foregut, midgut and hindgut through gastrulation and early somitogenesis. At E8.5, SOX17 is expressed in the midgut and hindgut dorsal regions but not in the foregut regions (Figure 3A) (Kanai‐Azuma et al. 2002). In E8.5 *Sox17* ^*Δdr/Δdr*^, SOX17 is not detected in the foregut, but it is expressed in the midgut and hindgut dorsal regions. However, the SOX17 staining signals in the midgut and hindgut dorsal regions in *Sox17* ^*Δdr/Δdr*^ embryos were weaker than those in WT embryos (Figure 3A).

At E9.5, SOX17 is primarily expressed in the posterior foregut and promotes the formation of the biliary bud, thereby initiating the appearance of the gallbladder (Figure 3B) (Spence et al. 2009). In *Sox17* ^*+/*−^ and *Sox17* ^*Δdr/Δdr*^ embryos, SOX17 was expressed in the biliary bud, but its staining signals were weaker than those of WT embryos. However, the reduced signal of SOX17 in *Sox17* ^*Δdr/Δdr*^ embryos was less striking than that in *Sox17* ^*+/*−^ embryos (Figure 3B).

We quantified the reduction level of SOX17 in *Sox17* ^*Δdr/Δdr*^ embryos using DAB staining to avoid the photobleaching of specific fluorescent signals during imaging and the IHC Profiler plugin in ImageJ (Varghese et al. 2014). This semiquantitative measurement was validated by comparing the intensity of SOX17 between E8.5 *Sox17* ^*+/*−^ and littermate WT embryos (Figure S3). Approximately 50% reduction in SOX17 signals was confirmed in the midgut, hindgut and blood vessels of *Sox17* ^*+/*−^ embryos compared with WT embryos. Subsequently, we applied this measurement to the E9.5 biliary bud. The intensity of the SOX17 signal in *Sox17* ^*Δdr/Δdr*^ biliary buds decreased to 80% of that in WT embryos. However, its reduction was less severe than that in *Sox17* ^*+/*−^ embryos, which decreased to nearly 40% of that in WT embryos (Figure 3C).

**FIGURE 4:**
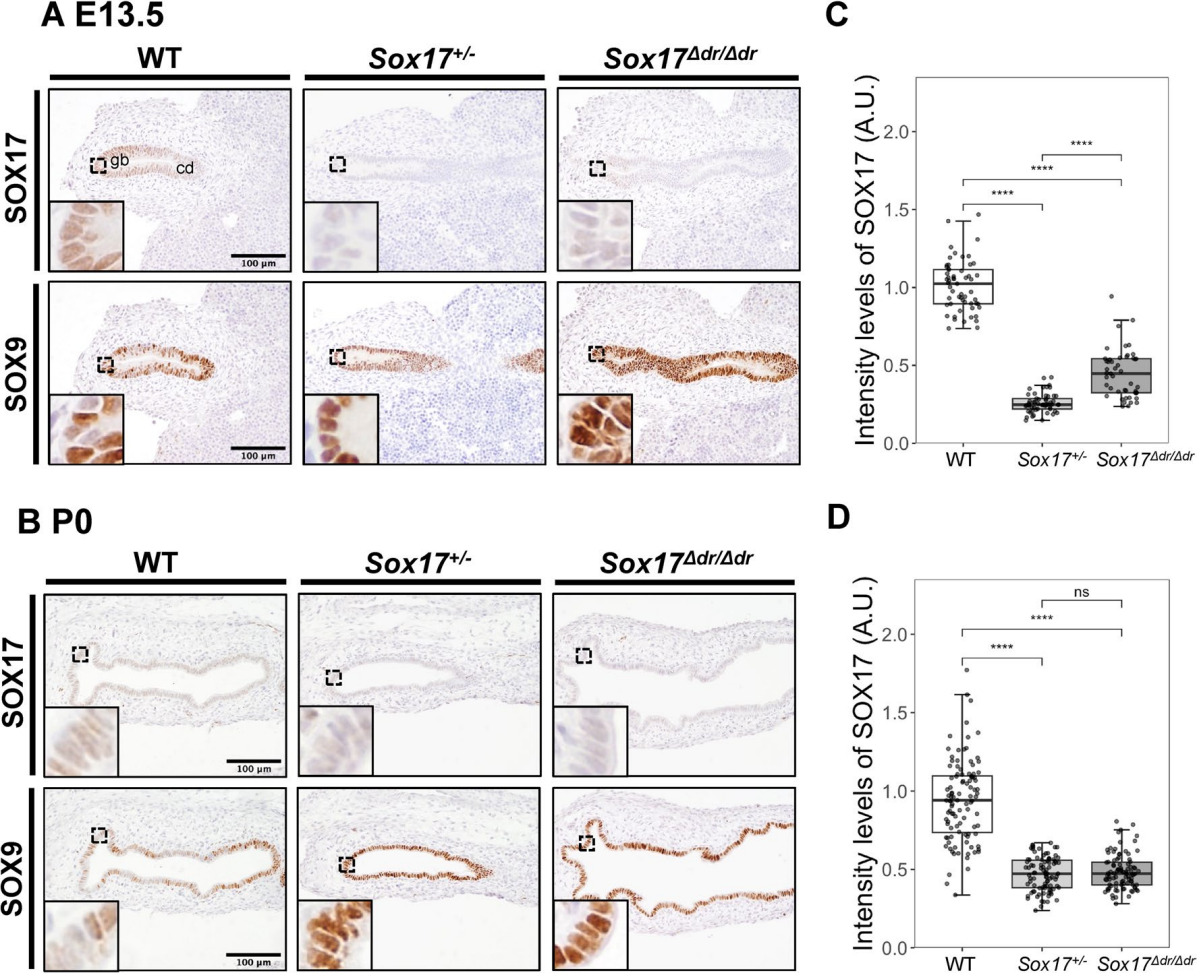
SOX17 and SOX9 expression in the embryonic and postnatal *Sox17* ^*Δdr/Δdr*^ gallbladder. (A, B) Immunostaining of the primordial gallbladder and P0 gallbladder from WT, *Sox17* ^+/−^ and *Sox17* ^*Δdr/Δdr*^ mice at E13.5. The insets show high magnification images of the boxed regions. Gb: gallbladder, cd: cystic duct. Scale bars: 100 μm. (C, D) Semiquantitative analysis of SOX17 staining intensity in the gallbladders of WT, *Sox17* ^+/−^ and *Sox17* ^*Δdr/Δdr*^ at E13.5 and P0. *n* = 4 for each group. The staining intensities were normalized to the average staining intensity of WT. ****p < 0.0001; ns: Not significant.

We further investigated SOX17 expression in biliary bud‐derived tissues at E13.5 and P0. SOX17 is highly expressed in the E13.5 embryonic gallbladder and cystic duct epithelium, and it is gradually enriched in the distal region of the embryonic gallbladder epithelium (Higashiyama et al. 2017). However, in *Sox17* ^*Δdr/Δdr*^ embryos, SOX17 expression levels were decreased in the E13.5 and P0 gallbladders (Figure 4A). The intensity level of SOX17 in the E13.5 and P0 *Sox17* ^*Δdr/Δdr*^ gallbladder epithelium was significantly decreased to nearly 50% of that of WT, and this reduction was consistent with that observed in P0 *Sox17* ^*+/*−^ mice (Figure 4C,D).

We examined the developmental stage of the gallbladder epithelium of E13.5 and P0 *Sox17* ^*Δdr/Δdr*^ embryos. Through biliary bud and gallbladder formation, the progenitor of the gallbladder epithelium initially expressed SOX17 and SOX9 (Saito, Kojima, and Takahashi 2013; Seymour et al. 2007). Alongside the gallbladder–cystic duct domain formation, the gallbladder epithelium consists of SOX17^high^SOX9^dim^ cells, whereas the cystic duct epithelium primarily consists of SOX17^dim^SOX9^high^ cells and a small population of SOX17^+^SOX9^+^ peribiliary glands (Higashiyama et al. 2017; Uemura et al. 2020). However, the expression levels of SOX9 remained high in the E13.5 and P0 *Sox17* ^*Δdr/Δdr*^ gallbladder epithelium, which is similar to those in *Sox17* ^*+/*−^ embryos, whereas few SOX9‐positive cells were observed in the WT gallbladder epithelium (Figure 4A,B) (Higashiyama et al. 2017). Morphological analysis showed that the P0 *Sox17* ^*Δdr/Δdr*^ gallbladder epithelium consisted of a pseudostratified columnar epithelium with epithelial folds, while the *Sox17* ^*+/*−^ gallbladder epithelium consisted of a single‐layered cuboidal epithelium with few epithelial folds showing severe hypotrophy (Figure 4A) (Higashiyama et al. 2017; Uemura et al. 2013). These results indicate that the gallbladder epithelium of *Sox17* ^*Δdr/Δdr*^ may undergo abnormal differentiation, although the gallbladder formed normally.

We also examined changes in SOX17 expression in other *Sox17*‐expressing organs at P0, including blood vessels, rete testes, oviducts and uteri (Hirate et al. 2016; Matsui et al. 2006; Uchida et al. 2022). *Sox17* ^*+/*−^ mice showed reduced SOX17 expression in the blood vessels, rete testes, oviducts, and uteri (Figure S4). However, the expression patterns and levels of SOX17 in the tissues of P0 *Sox17* ^*Δdr/Δdr*^ mice were similar to those of WT mice. These results indicate that the distal putative regulatory sequences of *Sox17* are crucial for the specific regulation of the SOX17 level in the embryonic gallbladder and for the proper differentiation of the gallbladder epithelium rather than for the formation of the gallbladder.

## 3 Discussion

### 3.1 Distal Putative Regulatory Sequences of *Sox17* Functions in Embryogenesis

Although *Sox17*‐null mice are embryonic lethal (Kanai‐Azuma et al. 2002), *Sox17* ^*Δdr/Δdr*^ mice survived to adulthood with a detectable SOX17 expression level (Figure 2). Therefore, although the distal regions of *Sox17* contribute to the regulation of SOX17 expression, they are not the only regulatory sequences for the critical developmental functions of *Sox17*. This finding is consistent with a previous report, that is, the proximal conserved putative promoter, rather than the near R1 distal putative regulatory sequence, plays a more crucial role in endoderm development (Trinh et al. 2022). A previous study on the evolutionarily conserved putative promoter region (near R1) reported that near‐R1 region deletion mice exhibited no gross morphological abnormalities in endoderm‐derived tissues with a modest increase in lymphatic vascular cell population (Trinh et al. 2022). In this study, the deletion of R1 and the rodent‐specific putative regulatory sequence R2 uncovered their potential function in the specific regulation of the gallbladder during embryogenesis. However, further investigation is necessary to determine which distal regulatory regions are essential for *Sox17* gene expression. In addition, several TCF/LEF‐binding elements and SOX‐binding elements are present in R1 and R2 (Engert et al. 2013). Further study on the mechanisms by which these elements are regulated during gallbladder development could elucidate the precise regulatory mechanism underlying *Sox17* gene expression.

### 3.2 SOX17 Expression Level and Timing Regulate Gallbladder Development

*Sox17* ^*Δdr/Δdr*^ mice exhibited a normal gallbladder size, whereas *Sox17* ^*+/−*^ mice exhibited gallbladder hypoplasia during development (Uemura et al. 2013) (Figure 2C). The biliary primordium and ventral pancreas emerge from the PDX1^+^SOX17^+^ common progenitor cells in the ventral foregut at E8.5. Then, PDX1^−^SOX17^+^ gallbladder progenitor cells were segregated from these common progenitor cells at E9.5 (Spence et al. 2009). The expression level of SOX17 decreased throughout gallbladder development (Figures 3 and 4). However, the reduction of SOX17 expression in the E9.5 *Sox17* ^*Δdr/Δdr*^ gallbladder primordia was mild compared with that observed in the *Sox17* ^*+/−*^ gallbladder primordium (Figure 3B,C). Early gallbladder formation may not rely on the distal regions of *Sox17*, and a mild reduction of SOX17 in the biliary primordium around E9.5 is insufficient to reduce the population of gallbladder and biliary progenitors, leading to gallbladder hypoplasia. The severe reduction of SOX17 expression level in the biliary system (gallbladder and cystic duct) of *Sox17* ^*Δdr/Δdr*^ embryos after the specified biliary primordium might affect the segregation of SOX9 and SOX17 expression and lead to the ectopic expression of SOX9 in the embryonic gallbladder.

In this study, the number of DBA‐ and PAS‐positive goblet‐like cells increased in P0 *Sox17* ^*Δdr/Δdr*^ gallbladders (Figure 2D,E). In humans, the increase of mucin and the appearance of goblet cells representing intestinal metaplasia in the gallbladder epithelium are often observed in patients with chronic gallbladder diseases, such as gallstones (Finzi et al. 2006; Vilkin et al. 2007; Yamagiwa and Tomiyama 1986). However, *Sox17* ^*Δdr/Δdr*^ mice grew normally up to adulthood. The origin of these PAS‐positive gallbladder epithelial cells requires further investigation.

These results suggest that the distal putative regulatory sequences of *Sox17* may regulate the expression level of SOX17 in a gallbladder‐specific manner during its development, leading to abnormal gallbladder epithelium differentiation. Therefore, further studies elucidating the respective functions of R1 and R2 will reveal species‐specific elaborate regulatory mechanisms underlying *Sox17* gene expression in embryonic development and homeostasis.

## 4 Experimental Procedures

### 4.1 Animals

The mice were maintained in designated animal rooms under a 12‐h light–dark cycle, and they were given ad libitum access to water and food at the University of Tokyo and Institute of Science Tokyo. *Sox17* ^*+/−*^ fetuses and pups were obtained from *Sox17* ^*+/−*^ male mice (B6 129SvJ mixed background) (Kim, Saunders, and Morrison 2007) mated with littermate WT females. C57BL/6JJcl and ICR mice were purchased from Clea Japan. The sex of fetuses was not considered in this study. All animal experiments in this study were performed in accordance with the guidelines of their Animal Care and Use Committee (approval ID: P23‐047, A2021‐192C14, and A2023‐102C3).

### 4.2 Generation of *Sox17* ^*Δdr*^ ^*/Δdr*^ Mice

The CRISPR/Cas9 system was introduced into fertilized mouse embryos (C57BL/6JJcl) through electroporation. The zygotes were prepared by in vitro fertilization and electroporated with 1 μg/μL Cas9 protein, 1 μg/μL tracrRNA and three 1 μg/μL crRNA targeting the distal region of *Sox17*, including R1 and R2 sequences (IDT) (Figure 2A), in Opti‐MEM media (Thermo Fisher Scientific) using CUY21EDIT II (BEX). After electroporation, the embryos were cultured in KSOM medium (ARK Resource) until the 2‐cell stage and transplanted into the oviducts of pseudopregnant ICR mice.

### 4.3 Genotyping

To extract genomic DNA, ear punches or embryonic yolk sacs were lysed in an alkaline lysis reagent (pH 12) at 95°C for 1 h, and an equal volume of neutralization reagent (pH 5) was added. The target region was amplified by PCR using GoTaq DNA polymerase (Promega) and the primers dr_F and dr_R1 to detect the *Sox17* mutation. The amplicons were column purified and sequenced by Sanger sequencing using the primer dr_R1. For genotyping of *Sox17* ^*Δdr/Δdr*^, the primers dr_F, dr_R1, and dr_R2 were used. 262 and 518 bp products were amplified from the mutant and WT alleles, respectively. For *Sox17* ^*+/−*^ genotyping, the primers SOX17+/−_F1, SOX17+/−_F2, and SOX17+/−_R2 (Kim, Saunders, and Morrison 2007) were used. 440 and 320 bp products were amplified from the mutant and WT alleles, respectively. The sequences of the primers used are listed in Table S2.

### 4.4 RNA Extraction, Reverse Transcription and PCR

The whole gallbladder and common bile duct were dissected from E13.5 WT and *Sox17* ^*Δdr/Δdr*^ mice to extract RNA. The samples were disrupted with a small bead in 1 mL of TRIzol reagent (Invitrogen) using TissueLyser LT (QIAGEN). Then, the samples were mixed thoroughly after adding 200 μL of chloroform. They were incubated for 2–3 min and centrifuged for 15 min at 12,000 × *g* at 4°C. The aqueous phase was transferred to an RNeasy spin column of the RNeasy Mini kit (QIAGEN), and total RNA was extracted. The extracted RNA was measured using Qubit (Invitrogen). Total RNA (100 ng) was used for reverse transcription with SuperScript IV VILO Master Mix (Thermo Fisher Scientific). RT‐qPCR was performed in Taqman Fast Advanced Mater Mix using Quant Studio 7 (Applied Biosystems). The primers and probes used are listed in Table S2.

### 4.5 Histology and Immunohistochemistry

All samples were fixed in 4% paraformaldehyde–PBS solution at 4°C overnight. On the following day, the samples were washed with PBS, dehydrated through an ethanol series, and embedded in paraffin. The paraffin serial sections (4 μm thickness) were subjected to conventional hematoxylin–eosin staining, AB‐PAS staining, and immunohistochemistry. For immunohistochemistry, each sample was incubated with primary antibodies or DBA lectin, incubated with secondary antibodies, and counterstained with Mayer's hematoxylin or DAPI. The antibodies used are listed in Table S2.

### 4.6 Comparative Genome Analysis

Genomic information was collected from the Ensembl database (https://www.ensembl.org/) and the UCSC genome browser (https://genome.ucsc.edu). Genome alignment was performed using mVISTA (Frazer et al. 2004) and SnapGene (www.snapgene.com). TimeTree (Kumar et al. 2017) was used to draw the evolutionary tree showing the relationships between species. Using the neighbouring genes of human *SOX17* as a reference, the upstream and downstream genes of *Sox17* were identified in various species. Details of the genome assemblies and genetic information surrounding *Sox17* in each species are provided in Table S1.

### 4.7 Imaging and Quantification

The samples were observed under a light microscope (BX43) or confocal laser microscope (TCS SP8). The immunohistochemistry signals were quantified using ImageJ (Schneider, Rasband, and Eliceiri 2012). In addition, the IHC Profiler plugin in ImageJ was used for color deconvolution (Varghese et al. 2014). Regions of interest containing only positively stained nuclei were manually selected, and the staining intensity was measured using the mean gray value parameter. The value of each measured cell is displayed as the reciprocal staining intensity (RSI), where RSI = 255—mean gray value (Cizkova et al. 2021). The RSI measured in *Sox17* ^*+/−*^ and *Sox17* ^*Δdr/Δdr*^ cells was divided by the average RSI of WT cells to obtain the relative signal intensity.

### 4.8 Statistical Analysis

Data were analyzed using the graphics and statistics program RStudio (RStudio Team 2020). Students' *t*‐test or ANOVA was used to determine the overall difference between two groups. The Tukey test was also to determine significant differences. *p < 0.05, **p < 0.01, ***p < 0.001, ****p < 0.0001, and NS *p* > 0.05.

## Author Contributions

**Shihan Zeng:** data curation, formal analysis, writing – original draft, investigation. **Ayaka Yanagida:** supervision, writing – original draft, writing – review and editing, investigation, funding acquisition. **Noriaki Ota:** investigation. **Mami Uemura:** funding acquisition, investigation. **Yoshikazu Hirate:** investigation. **Ryuji Hiramatsu:** writing – review and editing. **Naoaki Mizuno:** data curation, writing – review and editing. **Yoshiakira Kanai:** conceptualization, funding acquisition, supervision, methodology. **Masami Kanai‐Azuma:** supervision, funding acquisition.

## Conflicts of Interest

The authors declare no conflicts of interest.

## Supporting information


**Figure S1.** Comparison of two putative regulatory regions in mammals and birds.

**Figure S2.** Generation of (WT, *Sox17* ^*Δdr/Δdr*^) mice and their phenotype.

**Figure S3.** Validation of SOX17 expression quantification.

**Figure S4.** Expression analysis of SOX17 in the blood vessels and reproductive tract.

**Table S1.** Synteny block analysis data.

**Table S2.** Primer and probe sequencing and antibody information.

## Data Availability

The data that support the findings of this study are available from the corresponding author upon reasonable request.
