# Supplementary figures and images for: Identification of Gallbladder‐Specific Distal Regulatory Sequence of Murine Sox17
Source: Genes Cells. 2024 Dec 26;30(1):e13186. doi: 10.1111/gtc.13186 (PMC11671671; doi:10.1111/gtc.13186)

## Slide 1
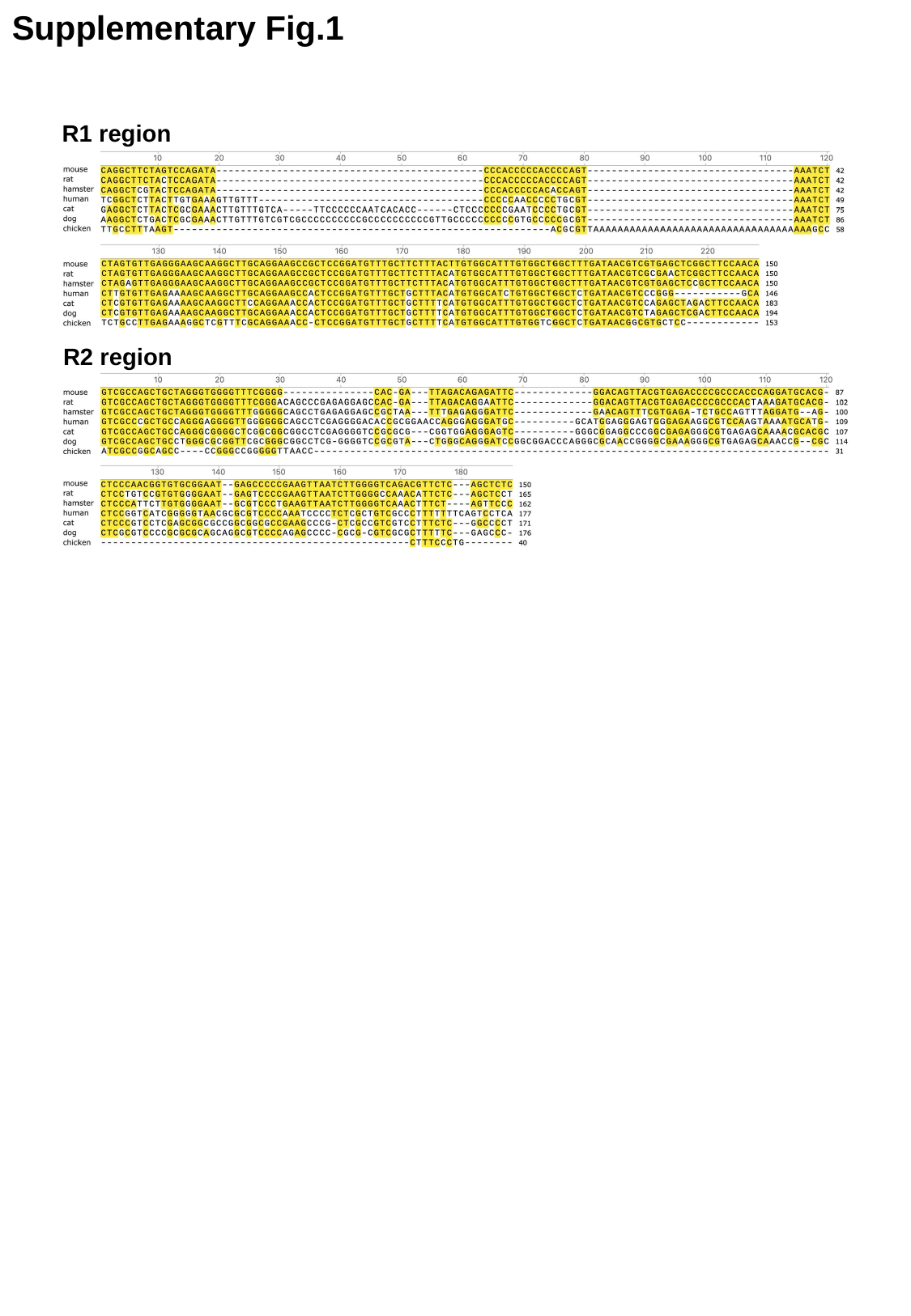

Supplementary Fig.1
R1 region
R2 region

Supplement: Supplementary file 1 — Figure S1. Comparison of two putative regulatory regions in mammals and birds. [file GTC-30-0-s005.pptx]

Supplementary Fig.3

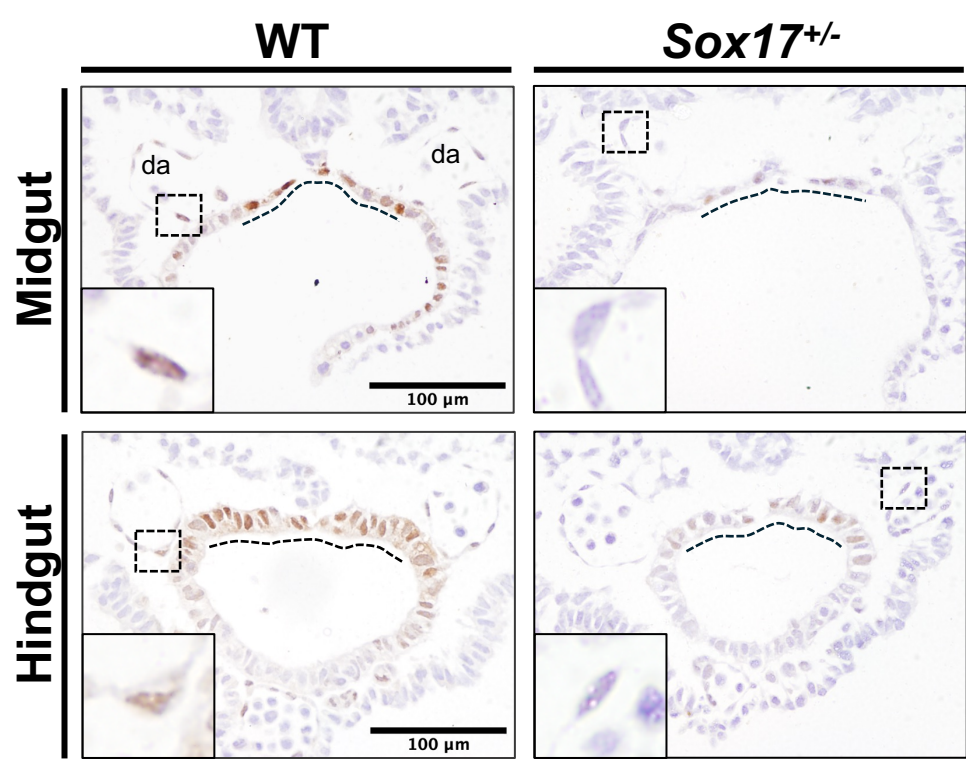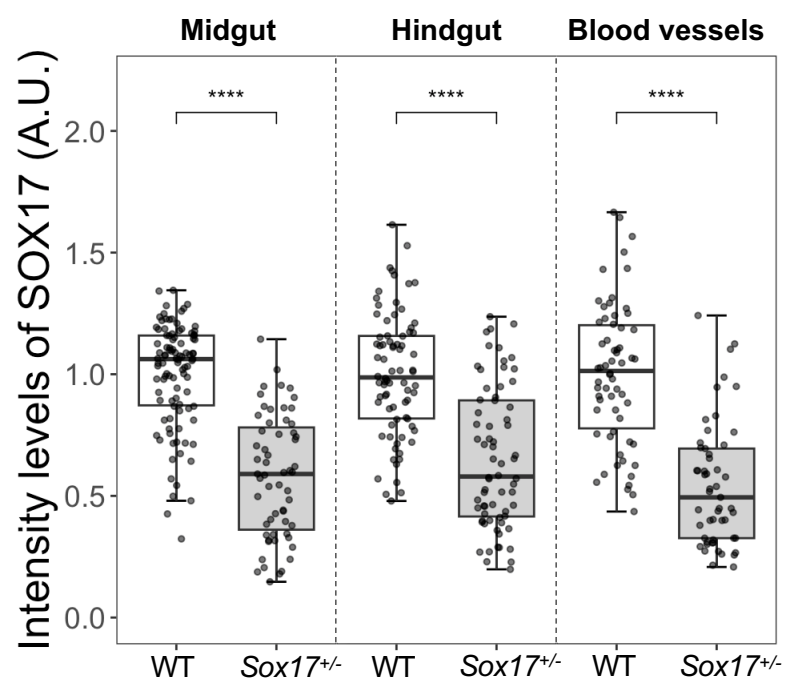

Supplement: Supplementary file 3 — Figure S3. Validation of SOX17 expression quantification. [file GTC-30-0-s003.pdf]
