# Supplementary material for: Identification of Gallbladder‐Specific Distal Regulatory Sequence of Murine Sox17
Source: Genes Cells. 2024 Dec 26;30(1):e13186. doi: 10.1111/gtc.13186 (PMC11671671; doi:10.1111/gtc.13186)
Supplement: Supplementary file 2 — Figure S2. Generation of (WT, Sox17 Δdr/Δdr ) mice and their phenotype. [file GTC-30-0-s002.pptx]

## Slide 1
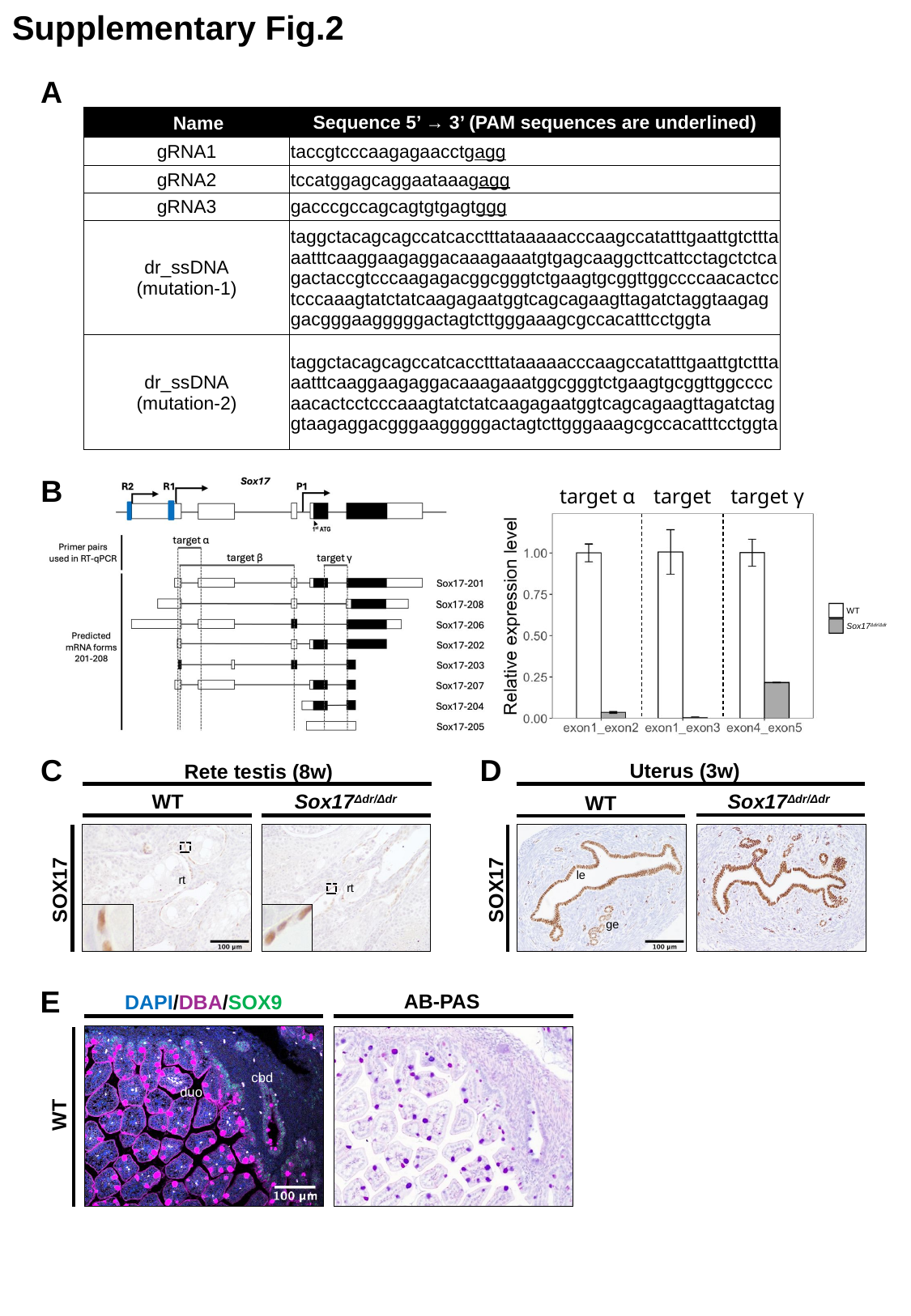

Supplementary Fig.2
A
| Name | Sequence 5’ → 3’ (PAM sequences are underlined) |
| --- | --- |
| gRNA1 | taccgtcccaagagaacctgagg |
| gRNA2 | tccatggagcaggaataaagagg |
| gRNA3 | gacccgccagcagtgtgagtggg |
| dr\_ssDNA (mutation-1) | taggctacagcagccatcacctttataaaaacccaagccatatttgaattgtctttaaatttcaaggaagaggacaaagaaatgtgagcaaggcttcattcctagctctcagactaccgtcccaagagacggcgggtctgaagtgcggttggccccaacactcctcccaaagtatctatcaagagaatggtcagcagaagttagatctaggtaagaggacgggaagggggactagtcttgggaaagcgccacatttcctggta |
| dr\_ssDNA (mutation-2) | taggctacagcagccatcacctttataaaaacccaagccatatttgaattgtctttaaatttcaaggaagaggacaaagaaatggcgggtctgaagtgcggttggccccaacactcctcccaaagtatctatcaagagaatggtcagcagaagttagatctaggtaagaggacgggaagggggactagtcttgggaaagcgccacatttcctggta |
B
target α
target γ
target β
WT
Sox17Δdr/Δdr
target α
target γ
target β
C
D
Uterus (3w)
Rete testis (8w)
WT
Sox17Δdr/Δdr
Sox17Δdr/Δdr
WT
le
rt
SOX17
SOX17
rt
ge
E
AB-PAS
DAPI/DBA/SOX9
cbd
duo
WT
