# Supplementary material for: Identification of Gallbladder‐Specific Distal Regulatory Sequence of Murine Sox17
Source: Genes Cells. 2024 Dec 26;30(1):e13186. doi: 10.1111/gtc.13186 (PMC11671671; doi:10.1111/gtc.13186)
Supplement: Supplementary file 4 — Figure S4. Expression analysis of SOX17 in the blood vessels and reproductive tract. [file GTC-30-0-s004.pptx]

## Slide 1
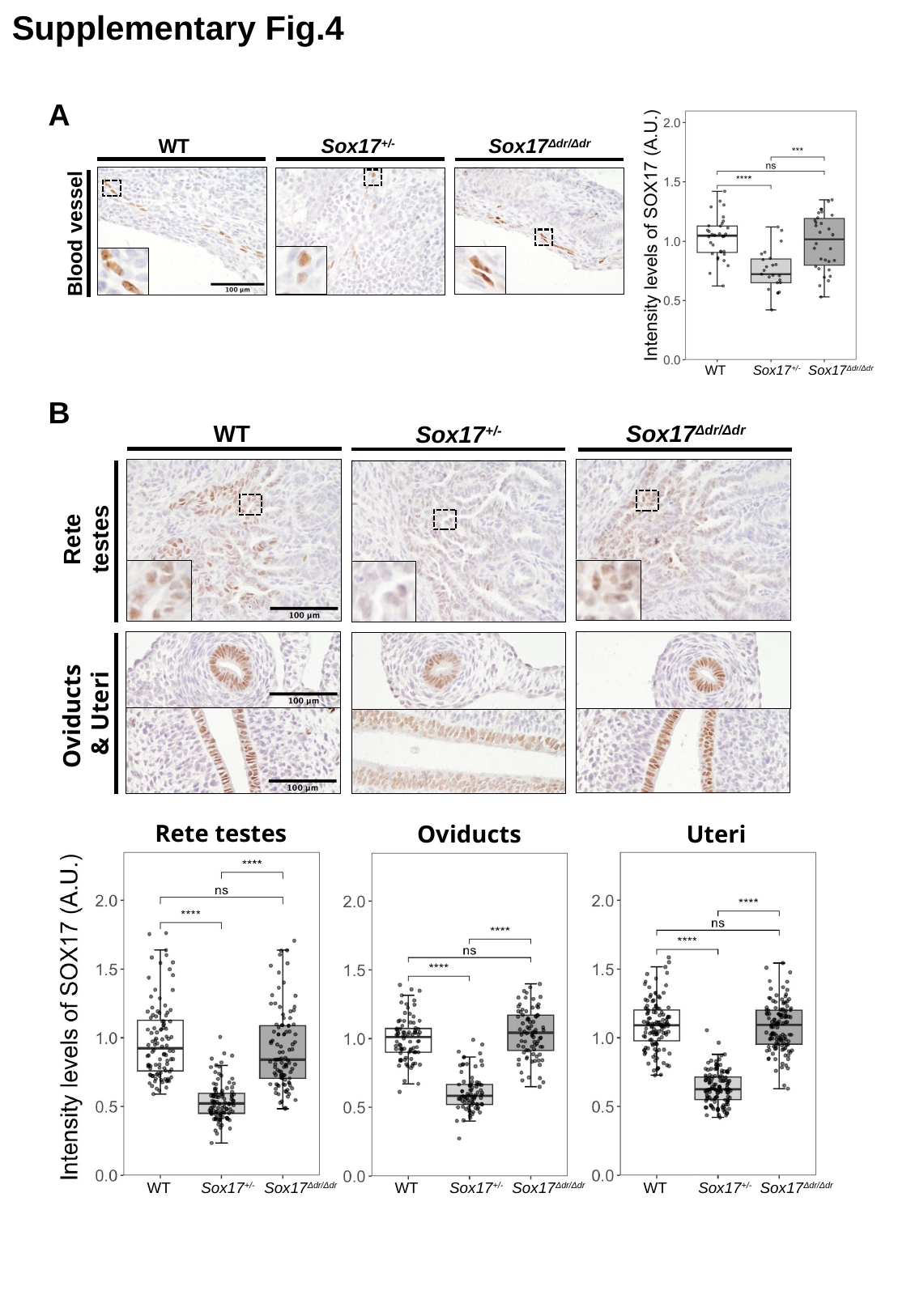

Supplementary Fig.4
A
WT
Sox17Δdr/Δdr
Sox17+/-
 Blood vessel
WT
Sox17+/-
Sox17Δdr/Δdr
B
Sox17Δdr/Δdr
WT
Sox17+/-
Rete testes
Oviducts
& Uteri
Rete testes
Oviducts
Uteri
WT
Sox17+/-
Sox17Δdr/Δdr
WT
Sox17+/-
Sox17Δdr/Δdr
WT
Sox17+/-
Sox17Δdr/Δdr
