# Supplementary material for: Identification of Gallbladder‐Specific Distal Regulatory Sequence of Murine Sox17
Source: Genes Cells. 2024 Dec 26;30(1):e13186. doi: 10.1111/gtc.13186 (PMC11671671; doi:10.1111/gtc.13186)
Supplement: Supplementary file 6 — Table S2. Primer and probe sequencing and antibody information. [file GTC-30-0-s006.pptx]

## Slide 1
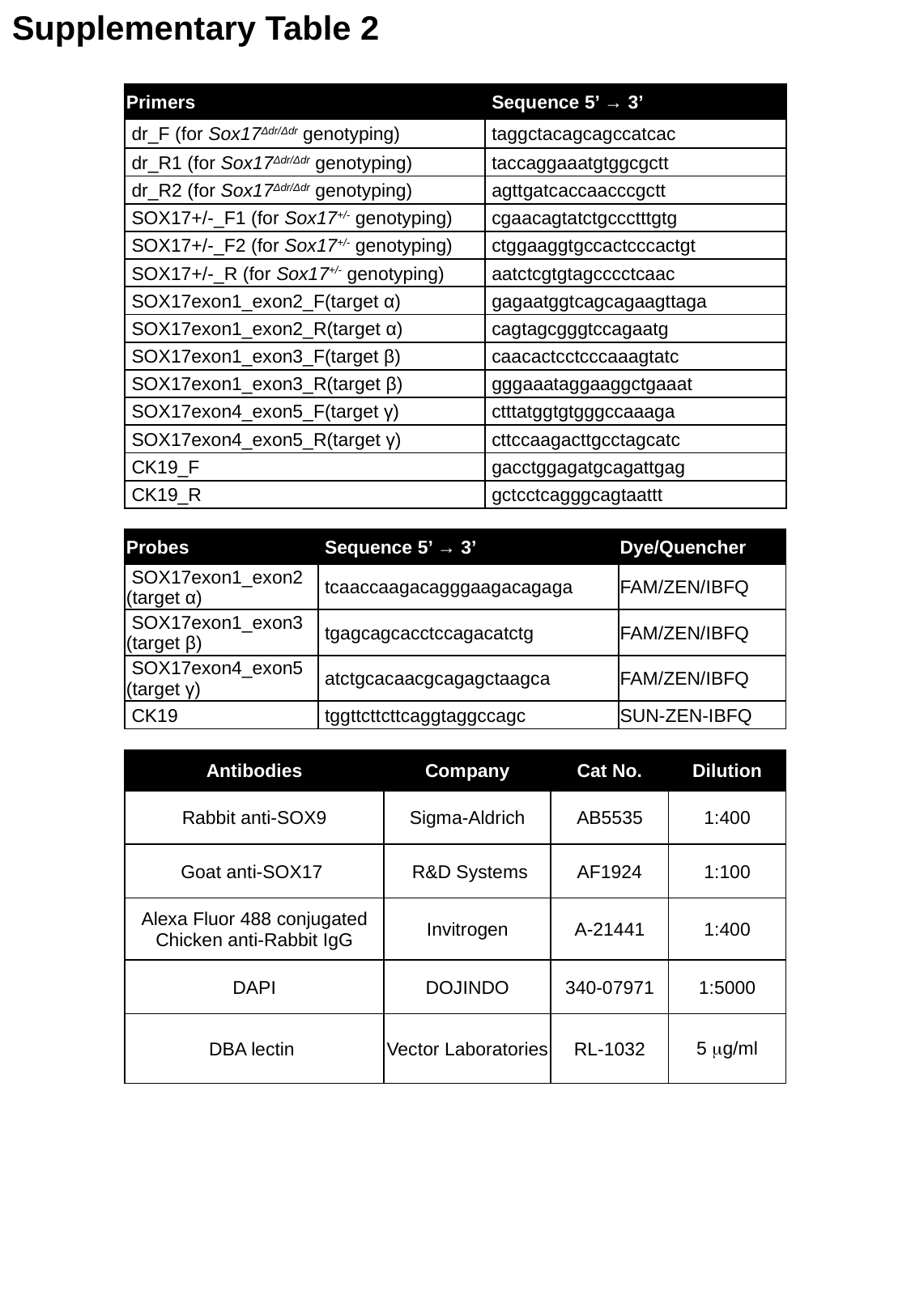

Supplementary Table 2
| Primers | Sequence 5’ → 3’ |
| --- | --- |
| dr\_F (for Sox17Δdr/Δdr genotyping) | taggctacagcagccatcac |
| dr\_R1 (for Sox17Δdr/Δdr genotyping) | taccaggaaatgtggcgctt |
| dr\_R2 (for Sox17Δdr/Δdr genotyping) | agttgatcaccaacccgctt |
| SOX17+/-\_F1 (for Sox17+/- genotyping) | cgaacagtatctgccctttgtg |
| SOX17+/-\_F2 (for Sox17+/- genotyping) | ctggaaggtgccactcccactgt |
| SOX17+/-\_R (for Sox17+/- genotyping) | aatctcgtgtagcccctcaac |
| SOX17exon1\_exon2\_F(target α) | gagaatggtcagcagaagttaga |
| SOX17exon1\_exon2\_R(target α) | cagtagcgggtccagaatg |
| SOX17exon1\_exon3\_F(target β) | caacactcctcccaaagtatc |
| SOX17exon1\_exon3\_R(target β) | gggaaataggaaggctgaaat |
| SOX17exon4\_exon5\_F(target γ) | ctttatggtgtgggccaaaga |
| SOX17exon4\_exon5\_R(target γ) | cttccaagacttgcctagcatc |
| CK19\_F | gacctggagatgcagattgag |
| CK19\_R | gctcctcagggcagtaattt |
| Probes | Sequence 5’ → 3’ | Dye/Quencher |
| --- | --- | --- |
| SOX17exon1\_exon2 (target α) | tcaaccaagacagggaagacagaga | FAM/ZEN/IBFQ |
| SOX17exon1\_exon3 (target β) | tgagcagcacctccagacatctg | FAM/ZEN/IBFQ |
| SOX17exon4\_exon5 (target γ) | atctgcacaacgcagagctaagca | FAM/ZEN/IBFQ |
| CK19 | tggttcttcttcaggtaggccagc | SUN-ZEN-IBFQ |
| Antibodies | Company | Cat No. | Dilution |
| --- | --- | --- | --- |
| Rabbit anti-SOX9 | Sigma-Aldrich | AB5535 | 1:400 |
| Goat anti-SOX17 | R&D Systems | AF1924 | 1:100 |
| Alexa Fluor 488 conjugated Chicken anti-Rabbit IgG | Invitrogen | A-21441 | 1:400 |
| DAPI | DOJINDO | 340-07971 | 1:5000 |
| DBA lectin | Vector Laboratories | RL-1032 | 5 mg/ml |
